# Antigen Cross-Presentation by Macrophages

**DOI:** 10.3389/fimmu.2020.01276

**Published:** 2020-07-08

**Authors:** Elke M. Muntjewerff, Luca D. Meesters, Geert van den Bogaart

**Affiliations:** ^1^Department of Tumor Immunology, Radboud Institute for Molecular Life Sciences, Radboud University Medical Center, Nijmegen, Netherlands; ^2^Department of Molecular Microbiology and Immunology, Groningen Biomolecular Sciences and Biotechnology Institute, University of Groningen, Groningen, Netherlands

**Keywords:** tumor immune responses, antigen cross-presentation, vacuolar pathway, macrophages, cytosolic pathway, T-cell activation, intracellular pathogens

## Abstract

The contribution of dendritic cell (DC) antigen cross-presentation to the activation of CD8^+^ T lymphocytes for immune defense against tumors, viruses, and intracellular pathogens has been recognized widely. Although originally thought to be an exclusive characteristic of DCs, recently also other immune cells, particularly macrophages, have been shown capable of cross-presentation. Here we provide an overview of *in vitro* and *in vivo* evidence on cross-presentation by macrophages. As we discuss, it is now firmly established that various types of tissue-resident macrophages are able to cross-present via similar cellular pathways as DCs. This is based on a wide range of antigens in macrophages from many different tissue origins such as blood, tumors, and lymphoid tissue. However, the physiological relevance of macrophage cross-presentation with potential contributions to activation of CD8^+^ T lymphocytes is still mostly unknown. While cross-presentation by various types of proinflammatory macrophages might be involved in cross-priming of naive CD8^+^ T lymphocytes, it might also be involved in local reactivation of memory and/or effector CD8^+^ T lymphocytes. Moreover, cross-presentation by anti-inflammatory macrophages could be related to immune tolerance. Because cross-presentation promotes the initiation and potentiation of antigen-specific CD8^+^ T lymphocyte responses, stimulating macrophages to cross-present antigen might be a promising strategy for antitumor or antiviral therapies.

## Introduction

Since the first evidence of their antigen cross-presenting capacities for activation of naive CD8^+^ T lymphocytes in 1976, dendritic cells (DCs) have been heavily studied for the mechanisms and physiological roles of cross-presentation (1). Antigen cross-presentation is crucial for the initiation of adaptive immune responses against cancer, viruses, and numerous other intracellular pathogens. During this process, antigen-presenting cells (APCs) present peptides derived from ingested antigens in their major histocompatibility complex class I (MHC-I) protein complex to naive CD8^+^ T lymphocytes. If the DCs also provide sufficient levels of costimulatory receptors (e.g., CD80, CD86) and cytokines [interleukin 12 (IL-12)], cross-presentation results in the activation of naive CD8^+^ T lymphocytes to effector cytolytic T cells in a process called cross-priming. Effector cytolytic T cells can induce apoptosis in infected or malignant cells (2, 3). Because DCs were assumed the only (or at least main) cross-presenting cells capable of cross-priming naive CD8^+^ T lymphocytes, by far most research has been focused on cross-presentation pathways in DCs. However, recently also macrophages have been shown to be capable of antigen cross-presentation, and this might have large implications for our understanding of CD8^+^ T lymphocyte responses. Therefore, the aim of this review is to provide an overview of the current studies covering the roles and mechanisms of antigen cross-presentation by macrophages.

### Role of Dendritic Cells and Macrophages in CD8^+^ T Lymphocyte Activation

During their lifetime, DCs can exist in two discrete stages: immature and mature. Immature DCs are overall considered to be better in endocytosis and protein processing, and their primary role is to sample antigen in the circulation and peripheral tissues. The recognition of antigen by pattern recognition receptors (PRRs), such as Toll-like receptors (TLRs), results in the maturation of the DCs. The resulting mature DC can migrate to the lymphoid organs, where it can activate antigen-specific CD8^+^ T lymphocytes by cross-presentation (4). Dendritic cells can be categorized into subsets based on their cross-priming ability and origin. Many studies are devoted to characterize the capacity of various DC subsets to cross-present to CD8^+^ T lymphocytes in mouse and human by, for example, the use of RNA sequencing and lineage tracing, which has been extensively reviewed elsewhere (2, 5, 6). Briefly, the main subsets in human are plasmacytoid CD123^+^CD303^+^CD304^+^ DCs (pDC) and conventional DCs (cDCs) including CD1a^+^CD11c^high^XCR1^−^BDCA-1^+^ (cDC2) and CD1a^−^CD11c^low^ CLEC9A^+^XCR1^+^BDCA-3^+^CD141^+^ (cDC1) (2, 5, 6). All these subsets have the capacity to cross-present, but because of the high expression of MHC-I pathway genes, cDC1s are considered as the most efficient cross-presenting cells in human (2, 5, 6). The same subsets are present in mice, but then based on expression of CD11c and MHC class II in combination with CD4, CD8α, CD11b, XCR1, CLEC9a, CD103, and CD205. The mouse classical DCs express either CD11b (equivalent to human cDC2) or CD8α, XCR1, CLEC9A (also known as DNGR1), and CD103 (equivalent to human cDC1), where the cross-presenting efficiency depends on the expression of CD8 (in lymphoid tissue), making the classical CD8^+^ DC the best mouse subset for activation of cytolytic T lymphocytes (2, 5, 6).

**Figure 1:**
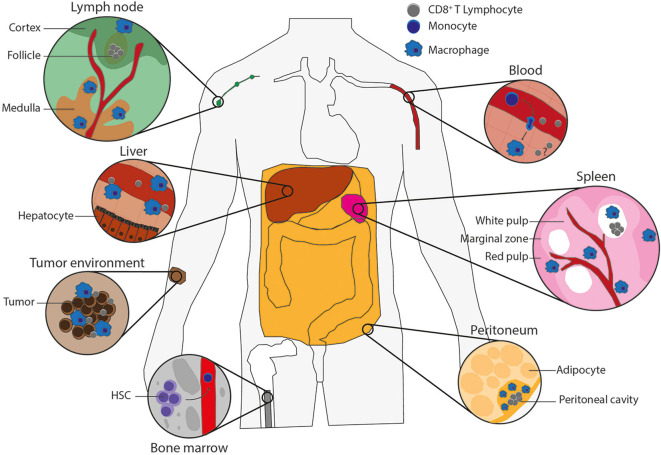
Macrophage types potentially capable of antigen cross-presentation. Populations of macrophages in various tissues of the body that can cross-present antigen and their potential interactions with CD8^+^ T lymphocytes. Lymph node: blood vessel (red), cortex and follicle (green) with CD8+ T lymphocytes (gray) in the T-cell zone and subcapsular macrophages on the edge (blue), medulla (brown) including medullary sinus macrophages, medullary cord macrophages and subcapsular macrophages (blue). Liver: blood vessel (red) containing CD8^+^ T lymphocytes (gray) and Kupffer cells (blue) on the edge, which are surrounded by hepatocytes. Tumor environment: tumor-infiltrating macrophages (blue) and CD8^+^ T lymphocytes (gray) in between tumor cells (brown). Bone marrow: hematopoietic stem cells (HSCs), which give rise to monocytes (dark blue) that leave the bone marrow by blood vessels (red). Blood: monocytes in the blood vessels (red) can extravagate through the vessel wall into tissues, where monocytes (dark blue) differentiate into macrophages (blue), and there could be memory T cells (gray) to reactivate upon infection. Spleen: the spleen consists of red pulp (pink), white pulp (soft pink) with follicles (white) containing CD8^+^ T lymphocytes (gray), and blood vessels (red). The red and white pulps are separated by the marginal zone. All contains macrophages (blue), including the marginal zone, which contains marginal metallophilic and marginal zone macrophages. Peritoneum: the adipocytes (soft yellow) surround the peritoneal cavity (yellow), which includes large and small peritoneal macrophages (blue) and CD8^+^ T lymphocytes (gray).

Tissue macrophages such as liver Kupffer cells, spleen red pulp, and large peritoneal macrophages develop during embryogenesis, where they originate from precursors in the yolk sac, fetal liver, and bone marrow (BM) [extensively reviewed in Perdiguero and Geissmann (7)]. These embryonically derived macrophages become tissue-resident macrophages that can propagate via self-renewal (7, 8). Later in life, the hematopoietic stem cells in the BM give rise to LY6C^+^ monocytes in mice or CD14^+^CD16^+^ monocytes in human, which can subsequently be recruited from the blood into the tissues, such as sites of infection or tumors, to promote either further inflammation or tissue repair (8–10). Upon arrival, these monocytes can differentiate into proinflammatory or anti-inflammatory monocyte-derived macrophages, depending on the PRR signaling, growth factors, and cytokines present in the tissue. The proinflammatory, also known as M1 or classically activated, macrophages are activated by signaling from PRRs or inflammatory cytokines [e.g., interferon γ (IFN-γ)] and express proinflammatory cytokines, IL-6, tumor necrosis factor α (TNF-α), IL-1β, and inducible nitric oxide synthase (6, 11). The anti-inflammatory, also called M2, proresolving or alternatively activated, macrophages are stimulated by IL-4 or IL-13 and express arginase 1, the mannose receptor CD206 and the IL-4 receptor α-chain (IL-4Rα). The main function of the proinflammatory macrophages is to eliminate tumors and pathogens by production of inflammatory cytokines and phagocytosis, whereas the main functions of the anti-inflammatory macrophages are tissue repair and homeostasis (12–14). When there is space in the tissue niche, blood-recruited monocytes might also differentiate into tissue macrophages (8). Both embryonically derived and monocyte-derived macrophages are able to sense and phagocytose tumor cells and pathogens (15). The precise phenotype and access to antigen of tissue-resident macrophages depend on the tissue, but especially spleen, lymph node, liver, and peritoneal macrophages (Figure 1) constantly encounter blood- or lymph-borne antigens, which means that they are ideally positioned for antigen uptake, and they are thus in principle well-positioned for cross-presentation to CD8^+^ T lymphocytes.

Although antigen cross-presentation by macrophages is less well understood than for DCs, it is increasingly clear that especially proinflammatory macrophages are capable of cross-presentation. The function of this cross-presentation is unclear, but evidence suggests it might be important for cross-priming of naive CD8^+^ T lymphocytes similar as in DCs. In addition, it might aid in the activation of memory CD8^+^ T lymphocytes, which are important in case of recurrent infections. Memory CD8^+^ T lymphocytes are present in tissues such as in the skin (16), where they might encounter macrophages. Additionally, inflammatory macrophages may cross-present to reactivate effector CD8^+^ T lymphocytes upon sustained infections in combination with production of IL-12 and IL-23, but independent of CD80, CD86, and CD28 costimulation (2). In contrast, cross-presentation by anti-inflammatory macrophages might have functions in immune tolerance against “self” proteins, commensal microbes, and food components, similar to the cross-presentation of immature DCs (17). The different roles of cross-presentation by macrophages possibly relate to the antigen source, type of macrophage, and location of the macrophage, as will be discussed below.

## Cross-Presentation by Various Macrophage Types

### Cross-Presentation by Splenic Macrophages

In the spleen, different subsets of macrophages can be distinguished based on their anatomical location and function (Figure 1). In mice, the red pulp macrophages (F4/80^high^CD68^+^VCAM1^+^CD11b^low^) are involved in iron metabolism and clearance of senescent erythrocytes. The white pulp macrophages (F4/80^low^CD68^+^) are specialized in phagocytosis, for instance, for clearance of apoptotic B cells, and for regulating immune responses against pathogens. The white and red pulps are separated by the marginal zone, and macrophages located in this area can recognize and clear blood-borne antigens. Marginal zone macrophages can be subdivided in the phenotypically and functionally different marginal metallophilic macrophages (F4/80^low^CD68^+^CD169^+^), involved in virus clearance, and marginal zone macrophages (F4/80^low^CD68^+^SIGN-R1^+^), involved in tolerance against apoptotic cells in blood (18). Given that both types of marginal zone macrophage encounter blood-borne antigens and the role of CD169^+^ macrophages in CD8^+^ T lymphocyte activation in the lymph nodes (see below), a role for these macrophages in CD8^+^ T lymphocyte activation in the spleen is likely (19, 20).

Evidence that splenic macrophages are capable of cross-presentation comes from the *in vitro* finding that isolated mouse splenocytes incubated with microspheres encapsulated with the model antigen ovalbumin could induce activation of ovalbumin-specific B3Z hybridoma cells (LacZ assay) (21). Macrophages might play a role in this CD8^+^ T lymphocyte activation, and it was not exclusively due to splenic DCs, because ovalbumin-specific T-cell activation (*in vivo* cytotoxicity assays) was still observed with splenocytes from mice that were depleted of CD11c^+^DCs (21). Moreover, splenocytes from mice depleted of both CD11c^+^DCs and CD11b^+^macrophages showed less T-cell activation compared to mice depleted of CD11c^+^DCs only, suggesting that macrophages can cross-present in the absence of DCs (21). However, the ovalbumin-containing microspheres used in this study were specifically designed for vaccination and thereby might enhance cross-presentation also in cell types that normally are not (or less) able of this process. Moreover, the physiological relevance of this finding is unclear, because CD11c and CD11b expression alone seems not sufficient to distinguish splenic macrophages and DCs (5). For instance, although depletion with CD11c will remove both CD11c^hi^CD11b^+^CD8α^−^MHCII^+^ and CD11c^hi^CD11b^−^CD8α^+^MHCII^+^ cDC subsets (22), the selection of CD11b is not sufficient to distinguish the various spleen macrophage populations because this would require selection on CD169 or SIGN-R1^+^ (5, 22). Moreover, this isolation method might result in contamination of the CD11c^+^ DC population with CD11c^int^F4/80^high^ red pulp macrophages (23).

Recently, these CD11c^int^F4/80^high^ red pulp macrophages became of interest because, similar to DCs, they express CD11c, and their location in the spleen allows them to acquire blood-borne antigens (23). Indeed, *in vitro* cross-presentation of fluorescently labeled ovalbumin by murine CD11c^int^F4/80^high^ CD4^−^CD8^−^CD11b^−^CD80^+^CD86^+^MHCII^+^Gr1^−^MARCO^−^ red pulp macrophages resulted in faster OT-I cell proliferation and higher expression of the T-cell activation markers granzyme-B, TNF-α, and production of IFN-γ than with the CD11c^high^CD8α^+^DEC205^+^ cDC1 subset (23). These OT-1 cells activated by the red pulp macrophages did not express activation markers CD127, KLRG1, and CX3CR1, suggesting they were so-called early effector cells, which do not develop into memory cytolytic T cells (23, 24). In contrast to the cDC1 cells, uptake of the model antigen ovalbumin by the red pulp macrophages relied on the mannose receptor CD206 (23). To address the role of cross-presentation by red pulp macrophages *in vivo*, mice negative for the transcription factor SpiC were studied (23). SpiC mice lack CD11c^int^F4/80^high^ red pulp macrophages, as SpiC is required for their development, whereas cDC1 DCs are unaltered because they rely on the transcription factor Batf3 (23, 25). Infection of SpiC knockout OT-1 mice with AdLGO adenovirus-expressing ovalbumin resulted in a higher initial viral load, whereas viral clearance by ovalbumin-recognizing OT-I cytolytic T cells was achieved after 10 days. In contrast, cDC1-deficient Batf3 knockout mice showed no changes in the initial viral load, but viral clearance was delayed until day 14 (23). In line with the KLRG1^−^CX3CR1^−^ phenotype of the cytolytic T cells induced by red pulp macrophages *in vitro, in vivo* reinfection experiments in SpiC knockout mice showed that red pulp macrophages are not essential for induction of memory cytolytic T-cell responses (23). Together, these findings suggest that cross-priming by red pulp macrophages is necessary to contain early viral spread by triggering a fast but short antiviral response, whereas the main function of cDC1 cells is cross-priming of cytolytic T cells for complete viral clearance and development of memory cytolytic T cells.

The cross-presentation capabilities of other splenic macrophages have also been investigated *in vivo*. First, in mice with melanoma that underwent thermal ablation, isolated CD11b^+^CD11c^−^CD45^+^ splenic macrophages were shown capable of cross-presentation as directly shown with a fluorescently labeled antibody recognizing MHC-I (H-2Kb-) loaded with the ovalbumin-derived epitope SIINFEKL (26). In addition, the CD8^+^ lymphocytes in the tumor were specific for the antigen as shown by MHC-I tetramers carrying this epitope (26). Second, increased numbers of CD169^+^F4/80^+^CD11b^+^CD45^+^ macrophages were observed in the treated tumor region and spleen, which might suggest a role for these CD169^+^ macrophages in CD8^+^ T lymphocyte activation for elimination of the tumor (26). In line with a role for macrophages in cross-presentation, depletion of all phagocytosing cells using clodronate liposomes showed that after 7 days, when the DCs already repopulate, whereas the spleen macrophages need 2 weeks, the CD11c^+^ DCs were unable to activate OT-I cells (IFN-γ, ovalbumin-specific cytotoxicity) with ovalbumin targeted by an antibody to the Siglec-1 receptor (CD169) *in vitro* (27). In contrast, direct targeting to CD8^+^ DCs via ovalbumin conjugated to an antibody recognizing CD205 resulted in efficient activation of CD8^+^ T lymphocytes *in vitro* (27), indicating that directly targeted antigen was cross-presented by the DCs.

However, a role for DCs in activation of cytolytic T cells by macrophages cannot be excluded, as the antigen might be transferred from the macrophages to the DCs (26). Thus, although, for instance ovalbumin targeted to the receptor Siglec-1 is ingested by marginal metallophilic macrophages, it might be subsequently transferred to CD8^+^ DCs for cross-presentation to CD8^+^ T lymphocytes (27). CD8^+^ DCs not only reside in the white pulp, but also locate the red pulp and marginal zone (28), and in principle they could encounter marginal metallophillic macrophages residing in the latter area. Migration of the DCs to the marginal zone might be important for antigen transfer, because treatment of mice with pertussis toxin, which blocks chemokine receptor–mediated migration, no longer resulted in OT-I CD8^+^ T lymphocyte proliferation using ovalbumin targeted to Siglec-1 (27). In contrast, CD8^+^ T lymphocyte responses after direct targeting of ovalbumin to CD8^+^ DCs via CD205 were unaffected by treatment with pertussis toxin (27). The CD169^+^ macrophages and CD8^+^ DCs have also been visualized in close interaction with each other using immunofluorescence microscopy and flow cytometry. In this case, the CD169^+^ macrophages were suggested to move to the CD8^+^DCs, although this was not specifically shown (29). Moreover, the specific antigen transfer seems to depend on the sialoadhesin CD169, which is involved in cell adhesion, because the use of Sn-KI mice, which expresses a mutated form of CD169, resulted in a reduced amount of antigen-positive CD8^+^ DCs (fluorescent ovalbumin) and a reduced amount of ovalbumin-specific IFN-γ-producing CD8^+^ lymphocytes (29). The immune stimulus of CD169^+^ macrophages is also important, because *in vivo* administration of liposomes targeted to CD169L, present on CD169^+^ macrophages, and containing ovalbumin and a TLR7 agonist induced expression of the activation markers CD86 and CD80 in the CD169^+^ macrophages and induced *in vivo* OT-I CD8^+^ T lymphocyte activation measured by proliferation, whereas only OT-II CD4^+^ T-cell responses were observed in absence of the TLR7 agonist (30). Altogether, it seems that murine CD169^+^ macrophages in the spleen contribute to cross-presentation either directly or by transferring antigens to CD8^+^ DCs in the spleen.

### Cross-Presentation by Lymph Node Macrophages

According to their anatomical location in the lymph nodes and expression of surface markers, lymph node macrophages can be divided into subcapsular sinus macrophages (SSMs; F4/80^−^CD169^+^), medullary sinus macrophages (MSMs; F4/80^+^CD169^+^), and medullary cord macrophages (MCMs; F4/80^+^CD169^−^) (31) (Figure 1). Many lymph node macrophages are directly exposed to lymph fluid (31), which in principle enables them to efficiently take up lymph-borne antigens to present to T cells (31).

*In vivo* evidence that F4/80^+^CD169^+^ MSMs and F4/80^+^CD169^−^ MCMs are capable of cross-presentation comes from the finding that only these cells can induce antitumor effects through stimulation of tumor-specific CD8^+^ T lymphocytes when targeted by a nanogel packed with tumor-specific synthetic long peptide antigen (LPA) and a TLR9 agonist (32). In this study, isolated F4/80^+^CD169^+^ MSMs, 18 h after injection of the nanogel, were most efficient at inducing LPA-recognizing DUC18 CD8^+^ T-cell responses *in vitro* measured by IFN-γ production and proliferation, which is possibly caused by a higher expression of CD80 and CD86 when compared to MCMs (32). Moreover, DCs and F4/80^−^CD169^+^ SSMs did not induce T-cell responses, and thereby, it was concluded that they were not essential for cytolytic T-cell activation, probably because they did not have access to the nanogel. Injection of clodronate-containing liposomes, which deplete macrophages but less DCs because they regenerate faster, blocked these antigen-specific CD8^+^ T lymphocyte response in the lymph node (32). However, the physiological relevance of this finding is unclear, given that a highly artificial antigen was used, and it was not confirmed whether *in vivo* the macrophages actually processed the peptide for cross-presentation, presented it directly on MHC-I, or transferred it to DCs as described for splenic CD169^+^ macrophages (27, 29).

Other *in vivo* evidence that cross-presentation by CD169^+^ macrophages might be important *in vivo* is the finding that mice specifically depleted of CD169^+^ SSM and MSM macrophages by induced expression of diphtheria toxin receptor (DTR) showed no OT-I CD8^+^ T lymphocyte responses upon injection with ovalbumin expressing apoptotic cells or tumors (33). Additionally, CD169^+^CD11c^+^ macrophages were better cross-presenters compared to CD169^+^CD11c^−^ macrophages and CD169^−^CD11c^+^CD8^+^ DCs, as shown by footpath stimulation with ovalbumin-expressing dead cells, followed by flow sorting of the DC and macrophage subsets and *in vitro* culturing with OT-I cells (proliferation, IFN-γ). Moreover, injection of CD8α^+^ DCs in CD169^−^-depleted mice did not increase OT-I CD8^+^ T lymphocyte responses (proliferation, IFN-γ) (33). Although this result at first glance seems strange given the essential role of CD8α^+^ cDCs in CD8^+^ T lymphocyte activation (2), DCs are mostly located in the T-cell zone upon arrival in the lymph node, where the antigens might possibly not be well accessible to the DCs, whereas the sinus location of the macrophages is beneficial for the antigen sampling (12). Additionally, microscopy revealed that CD169^+^ macrophages and ovalbumin-specific OT-I CD8^+^ T lymphocytes are in close contact already at 4 h after antigen challenging, suggesting that the CD8^+^ T lymphocytes relocalize to the sinus to be activated by antigen cross-presentation and then return to the T-cell zone for proliferation (33, 34). Thus, CD169^+^ sinus macrophages might well activate CD8^+^ T lymphocytes in the sinus, which would suggest that they are capable of cross-presentation. This might explain the physiological relevance of these macrophages and their location in the sinus and not in the T-cell zone (Figure 1). Another option is that the CD169^+^ SSM and MSM might transfer the antigen to DCs by CD169 to contribute to CD8^+^ T lymphocyte activation, similar to CD169^+^ macrophages in the spleen (see above) (27, 29).

A role for CD169^+^ macrophages in cross-priming is in line with *in vivo* mouse experiments with subcutaneous injection of antibodies targeting different receptors and complexed with the model antigen ovalbumin fused to immunoglobulin G–binding domains of protein G (35). In this study, it was found that targeting to CD11c, CD40, and TLR2 (all present in DCs) resulted in the most efficient *in vivo* cross-priming of OT-I T cells, whereas targeting to CD207 (expressed strongly by Langerhans cells) or CD169 (expressed by SSMs) still resulted in stronger cytolytic T-cell responses than ovalbumin alone (i.e., without antibody targeting). In addition, microscopy showed that the protein complexes were ingested not only by CD35^+^ follicular DCs, but also by CD169^+^ cells located in the marginal zone (35).

The cross-presentation capabilities have also been studied for macrophages from the tonsils. Although CD11c^+^HLA-DR^+^CD14^+^ macrophages isolated from human tonsils efficiently ingested fluorescently labeled necrotic cells, *in vitro* activation (IFN-γ) of the MelanA-recognizing LT12 CD8^+^ T-cell clone pulsed with a long peptide fragment of MelanA was much less efficient for macrophages than for CD11c^+^HLA-DR^+^CD14^−^BDCA1^+^ cDC2, CD11c^+^HLA-DR^+^CD14^−^BDCA3^+^ cDC1, and CD11c^−^HLA^−^DR^+^ pDC tonsil DCs (36). Also, the *in vitro* cross-presentation of NS3 protein from hepatitis C to NS3 antigen-specific CD8^+^ T-cell clones (IFN-γ) was less efficient for CD11c^+^HLA-DR^+^CD14^+^ tonsil macrophages than for these DC subsets (36). These findings show that although tonsil macrophages might in principle be able to cross-present antigens, they are less capable than the major DC subsets.

### Cross-Presentation by Liver Macrophages

The macrophages in the liver are called Kupffer cells. This very heterogeneous cell population (37) plays a major role in the clearance of gut-derived antigens and pathogens from the blood, making them in principle ideally positioned for cross-presentation to blood CD8^+^ T lymphocytes. However, studying the Kupffer cells is challenging because of loss of cells during isolation and lack of consistent cell-membrane markers for sorting (37).

Murine hepatic cells cultured *in vitro* with the model antigen ovalbumin could induce proliferation of ovalbumin-recognizing T_H_1 HTL clone cells but not T_H_2 HTL cells (38, 39). Although this cell mixture included Kupffer cells, the contribution of other hepatic cells, such as liver resident DCs, liver endothelial cells, and hepatocytes, cannot be excluded. Indeed, a recent side-by-side comparison of ovalbumin-pulsed hepatocytes, Tie-2^+^CD11b^low^ liver endothelial cells, and CD11b^+^F4/80^+^ Kupffer cells showed that all these cell types can induce OT-I CD8^+^ T lymphocyte proliferation *in vitro* with similar efficiency as splenic CD11c^+^DCs (40). However the physiological role in the initiation of cytolytic T-cell responses by liver cells is unclear, because the levels of cytolytic T-cell activation markers (CD44, CD25) and IFN-γ production were lower in the proliferated OT-I CD8^+^ T lymphocytes upon stimulation by the liver cells than upon stimulation with CD11c^+^ DCs (40).

Interestingly, Kupffer cells seem to suppress immune responses for immune tolerance as seen in murine transplantation studies by rejection of allogeneic liver transplants (41). Injection of Carboxyfluorescein succinimidyl ester (CFSE)-labeled OT-I CD8^+^ lymphocytes in the portal vein and intraperitoneal injection of ovalbumin peptide resulted in retention of activated OT-I CD8^+^ cells (proliferation and CD45 expression) in the liver, followed by their deletion, probably by apoptosis as shown by DNA fragmentation (42). This apoptosis seems to be dependent on interactions with the Kupffer cells because CSF-1–deficient mice, which have no mature developed tissue macrophages because CFS-1 is essential for their differentiation, resulted in an increased presence of activated OT-I CD8^+^ lymphocytes (proliferation and CD45 expression) (42). Thus, it seems that Kupffer cells are capable of cross-presentation, and although the physiological relevance of this is still unclear, this might play a role in immune tolerance.

### Cross-Presentation by Tumor-Infiltrating Macrophages

The immune system can either combat cancer by immunosurveillance where the cancer is recognized and cross-presented by APCs (43) or promote tumor progression via (in)direct suppression of CD8^+^ T lymphocytes and other immune cells (43, 44). When directly comparing cross-presenting capacities of DCs and primary macrophages isolated from human peritoneal tumor ascites *in vitro*, it was found that HLA-DR^+^CD11c^+^CD1c^−^CD16^+^ tumor macrophages are even more effective than HLA-DR^+^CD11c^+^CD1c^+^CD16^−^ DCs at cross-presentation to the LT12 CD8^+^ T-cell lines (IFN-γ) recognizing the tumor-specific antigen MelanA, which does not require costimulation for activation (45). Moreover, thermal ablation of ovalbumin-expressing melanoma in mice resulted in increased cross-presentation of ovalbumin-derived epitopes by intratumoral CD11b^+^CD11c^−^ macrophages as directly measured by an antibody that recognizes MHC-I loaded with SINFEKL (26). Additionally, this cross-presentation was concomitant with an increased presence of ovalbumin-specific CD8^+^ T lymphocytes (26). Although these findings support a role for macrophages in ovalbumin-specific CD8^+^ T-cell responses, evidence shows that DCs are essential in the initial activation of the naive CD8^+^ T lymphocytes.

Although human ascites monocyte-derived CD1a^+^CD16^+^ macrophages efficiently presented a MelanA epitope to the LT12 CD8^+^ T-cell clone *in vitro*, cross-priming of allogeneic naive cytotoxic CD8^+^ T cells (proliferation and expression of granzyme A, perforin, IFN-γ) was only observed by ascites monocyte-derived DCs and not ascites monocyte–derived macrophages (45). The reason for this is that the ascites monocyte-derived macrophages lacked expression of costimulatory signals such as CD80 and CD86 and produced almost no IL-12, whereas these were expressed by the ascites monocyte-derived DCs (45). These findings indicate that although tumor-infiltrating macrophages are capable of cross-presentation, they do not provide the costimulatory signals required for cross-priming of cytolytic T cells.

More indirect evidence on cross-presentation by tumor-infiltrating macrophages was obtained in a microscopy study in mice showing that migrating OT-I CD8^+^ T lymphocytes in ovalbumin-expressing tumors can have long interactions with F4/80^+^ macrophages, which might suggest activation by cross-presentation (46). Furthermore, CD8^+^ T lymphocytes might be activated for virus peptide by macrophages, because tumors in mice caused by injected fibrosarcoma cells transfected with lymphocytic choriomeningitis virus [MCA102(gp33)] were infiltrated by CD8^+^ T lymphocytes, a few CD11c^+^ DCs and a high number of CD11b^+^ macrophages (44). However, these tumor-infiltrating CD8^+^ T lymphocytes exhibited a highly activated phenotype (upregulated CD25, CD44, and CD69 and down-regulated CD62L expression) but lacked effector cell function, measured by a killing assay, suggesting modulation of the CD8^+^ T lymphocytes by the tumor environment. The tumor-infiltrating CD11b^+^ macrophages were also able to efficiently cross-present to tumor infiltrating CD8^+^ T lymphocytes (proliferation, IFN-γ, cytolytic activity) for gp33 *in vitro* (44). However, the costimulatory molecules CD80, CD86, and ICAM-1 were not expressed in these macrophages, which could explain the observed loss of the killing by the CD8^+^ T lymphocytes (44).

## Cellular Pathways of Cross-Presentation by Macrophages

In DCs, antigen cross-presentation can be the result of two distinct cellular pathways: the cytosolic and vacuolar pathway (2, 47). In the cytosolic pathway, proteins are first transported from the lumen of the endosomal compartment to the cytosol for degradation by the proteasome. Subsequently, the derived peptides can be processed via the MHC-I presentation pathway. The peptides are relocated via the transporter associated with antigen presentation (TAP) into the endoplasmic reticulum (ER), where they are processed by ER aminopeptidases, or brought back into the antigen-containing endosomes to be processed by insulin-regulated aminopeptidase. The loading of peptides on MHC-I occurs within these compartments (2, 17). The other main cross-presentation pathway is the vacuolar pathway, where proteins are processed by endosomal/lysosomal proteases, such as cathepsin S, and loaded on MHC-I within the endosomal/lysosomal compartments (2, 17). As we will discuss below, evidence suggests that the vacuolar pathway seems to be predominantly used by macrophages, although they might be able to also cross-present via the cytosolic pathway.

### Bone Marrow and Blood Monocyte-Derived Macrophages Cross-Present via the Vacuolar Pathway

Monocytes derived from hematological precursors in the BM can migrate via the blood stream to other tissues, where they can differentiate into macrophages to perform tissue specific functions, eliminate pathogens, or restore tissue homeostasis (48). Therefore, for research on mouse macrophages, stem cells are frequently isolated from the BM and differentiated into macrophages *in vitro*. For research on human macrophages, CD14^+^monocytes are often isolated from the blood. Both are then differentiated into macrophages *in vitro*, either by the use of macrophage colony-stimulating factor (M-CSF), which results in a homogenous macrophage population, or by the use of granulocyte–macrophage (GM)-CSF, which results in a cell population reflecting resident macrophages (6). However, GM-CSF is also used to differentiate DCs from monocytes; therefore, the macrophage culture might contain DCs (5, 6). Thereby, it should be kept in mind that *in vitro*–cultured monocyte-derived macrophages and monocyte-derived DCs have similarities (5, 6) and that both *in vitro* BM-derived and monocyte-derived macrophages and DCs are artificial cell populations that might not be identical to their *in vivo* murine and human counterparts (Figure 1).

The cross-presenting capacities of BM macrophages seem to be higher than BM DCs when tested side-by-side using liposomes encapsulating a mixture of the model antigen ovalbumin and the pore-forming protein sticholysin II. Macrophage colony-stimulating factor–differentiated BM macrophages were better in activating the ovalbumin-recognizing B3Z CD8^+^ T-cell line (which does not require costimulation) than GM-CSF–differentiated BM-derived DCs *in vitro* (49). This could be explained by the lower ability of the DCs to internalize the antigen-containing liposomes (49). Furthermore, inhibitors of the lysosomal proteases cathepsins and leupeptin resulted in a reduced efficiency of B3Z T-cell activation by the BM macrophages, whereas a proteasome inhibitor (epoxomicin) had no effect (49). This suggests that ovalbumin is processed for cross-presentation in lysosomes instead of the cytosol and therefore that cross-presentation occurs via the vacuolar pathway in BM-derived macrophages.

In line with the conclusion that BM-derived macrophages cross-present via the vacuolar pathway, the proteasome inhibitor lactacystin did not inhibit cross-presentation of fluorescein isothiocyanate–labeled ovalbumin peptide complexed with heat shock protein to CD8OVA1.3 T hybridoma cells (colorimetric bioassay for IL-2) in M-CSF–differentiated BM macrophages, activated 48 h with IFN-γ, whereas there was only a slight reduction in activation when TAP-deficient macrophages were used (50). In contrast to BM macrophages, proteasome inhibition resulted in a marked reduction of CD8OVA1.3 T hybridoma cell activation by GM-CSF–differentiated BM DCs (50), suggesting that these macrophages and DCs use different pathways for cross-presentation. Final evidence that BM macrophages employ the vacuolar pathway for cross-presentation comes from the finding that cross-presentation of ovalbumin to B3Z T cells by MAC-1^+^ BM macrophages was reduced by a peptide aldehyde inhibitor that also inhibits lysosomal proteases (LLnL), whereas a proteasome inhibitor (LLM) had no effect and resulted in normal B3Z T-cell activation (51). Thus, whereas BM DCs can use the cytosolic route of cross-presentation, antigens seem to be predominantly cross-presented by BM macrophages via the vacuolar pathway. This would explain why BM macrophages have lower expression and activity of the NADPH oxidase NOX2, which is essential for cross-presentation via the cytosolic pathway (52, 53).

Fewer data are available on cross-presentation mechanisms in human monocyte-derived macrophages. They can also cross-present via the vacuolar pathway similar to murine BM macrophages, because an *in vitro* study showed that the proteasome inhibitor lactacystin did not impair cross-presentation by human blood monocyte-derived CD1a^+^CD16^+^ macrophages of MelanA antigen to CD8^+^T-cell LT12 clones (IFN-γ), whereas lysosomal cysteine protease inhibition (with a pan-cathepsin inhibitor) impaired activation of this T-cell line (45). These results support that monocyte-derived macrophages can cross-present via the vacuolar pathway, which might explain why lysosomal proteases are expressed in higher levels in human monocyte-derived CD1a^+^CD16^+^ macrophages compared to monocyte-derived CD1a^+^CD14^+^ DCs (45). However, for various HIV-1 epitopes, evidence suggests that monocyte-derived macrophages can cross-present via both the cytosolic and vacuolar pathways. Proteasome inhibition by MG132 or epoxomicin did not completely block activation (IFN-γ) of epitope-specific CD8^+^ T lymphocyte clones by lipopolysaccharide (LPS) and R848 matured monocyte-derived macrophages, suggesting both proteasome-dependent and -independent processing, although inhibition of lysosomal cysteine proteases by E64 did not affect cross-presentation (54). In line with this notion that human monocyte-derived macrophages might cross-present via both the cytosolic and vacuolar pathways, similar to DCs, is that the difference in expression levels of NOX2, required for the cytosolic route of cross-presentation, is less clear for human monocyte-derived macrophages and DCs (55) than for murine BM macrophages and BM DCs (52).

Besides the macrophage types described so far, a new murine F4/80^+^ subset of APCs was described containing characteristics of both macrophages (CD64^+^MERTK^+^) and cDC2s (CD11c^hi^MHCII^hi^CD11b^+^CD24^+^CD64^+^CD169^+^), while being from mouse monocyte origin (56). These hybrid DC macrophages have been found in multiple tissues including lymph node and spleen, and they seem increased in the tumor microenvironment. Supporting a functional role in the tumor environment, these cells are efficient at B16 tumor cell uptake (GFP-labeled), and *in vivo* blocking of CSF1R-positive cells (blocking most macrophages, including the new DC macrophage hybrid subset) significantly decreased antigen cross-presentation to ovalbumin-specific OT-I CD8^+^ T lymphocytes (proliferation) (56). Moreover, when isolating these DC macrophage hybrid cells from lymph nodes of mice inoculated with ovalbumin-expressing cancer cells and *in vitro* culturing them with OT-I CD8^+^ T lymphocytes, efficient T-cell proliferation was observed (56).

### Splenic Red Pulp Macrophages Cross-Present via the Cytosolic Pathway

As discussed above, both *in vitro* and *in vivo* evidence indicates that murine CD11c^int^F4/80^high^ red pulp macrophages from spleen are capable of cross-presentation (23). At least for the model antigen ovalbumin, evidence shows that they process this antigen via the cytosolic pathway. Following its uptake via the mannose receptor CD206, microscopy showed that fluorescently labeled ovalbumin colocalizes with the mannose receptor to early endosomes but not to late endosomes in CD11c^int^F4/80^high^ red pulp macrophages (23). *In vitro* cross-presentation to OT-I T cells was almost completely blocked by the TAP-inhibitor UL49 and the proteasome inhibitor epoxomicin (23), indicating that red pulp macrophages cross-present via the cytosolic pathway.

### Peritoneal Macrophages Cross-Present via Both Cellular Pathways

There are two subsets of peritoneal macrophages: large peritoneal macrophages (F4/80^high^, MHC-II^low^) that play a role in maintaining homeostatic conditions in the peritoneal cavity and represent an anti-inflammatory type, and small peritoneal macrophages (F4/80^low^, MHCII^high^), which are important during inflammation (57) (Figure 1). The large peritoneal macrophages express higher levels of TLR-4 and costimulatory molecules (CD80/CD86/CD40). Likely, both peritoneal macrophage types can cross-present (58–61), but there is contrasting evidence whether peritoneal macrophages use the vacuolar or cytosolic pathway. The yield of macrophages from the peritoneum is low; therefore, mice are often prestimulated with thioglycolate, which recruits immature macrophages into the peritoneum. Thioglycolate isolated peritoneal macrophages resemble mostly small peritoneal macrophages, but have an atypical morphology and function that is not consistent with the phenotype of tissue macrophages, but more with monocyte-derived macrophages (62). Moreover, during the isolation, they can come in contact with LPS from the used broth, which is ingested by the macrophages, and as a result, they are able to respond to IFN-γ priming without any other stimulation (63). A better option for isolation of peritoneal macrophages for cross-presentation and phagocytosis studies is called Bio-Gel–elicited macrophage isolation, which makes use of Bio-Gel beads that cannot be phagocytosed (63).

The angiotensin-converting enzyme (ACE) 10/10 mice overexpress the peptidase ACE, which is a peptidase normally located within the ER and involved in generation of MHC-I epitopes. Angiotensin-converting enzyme is also involved in cross-presentation probably following its transfer antigen-containing phagosomes or endosomes (2, 61). The injection of ovalbumin-pulsed peritoneal macrophages isolated from ACE 10/10 mice (using thioglycolate) and ovalbumin-specific OT-1 CD8^+^ T lymphocytes into a wild-type mouse resulted in proliferation of the CD8^+^ T lymphocytes (61). Moreover, upregulation of the CD8^+^ T lymphocyte activation marker CD69 occurred faster with peritoneal macrophages isolated from the ACE 10/10 mice than with macrophages isolated from wild-type mice (61). Although these studies rely on overexpression of ACE, these findings indicate that peritoneal macrophages in principle can cross-present antigen (61). Moreover, as ACE is potentially transferred from the ER to the lumen of antigen-containing endosomes (61), these findings suggest that peritoneal macrophages can cross-present via the vacuolar pathway.

More direct evidence that peritoneal macrophages can cross-present comes from the finding that a murine peritoneal macrophages cell line that recombinantly expresses IFN regulatory factor 7 was able to induce activation of ovalbumin-specific OT-I CD8^+^ T lymphocytes *in vitro* (IL-2 production and CD40, CD80, and CD86 expression) (58). This activation could be inhibited by a proteasome inhibitor (lactacystin) (58), suggesting that the cytosolic pathway was used for cross-presentation of the antigen. Also the uptake by peritoneal macrophages of *Escherichia coli* in which ovalbumin was expressed could trigger activation (IL-2 production) of the ovalbumin-recognizing B3Z CD8^+^ T-cell line (60). These ingested *E. coli* bacteria remained in phagosomes that fused with lysosomes (60), suggesting processing of the bacteria for cross-presentation via the vacuolar pathway in this case.

Murine peritoneal macrophages also cross-presented virus-like particles containing the immunodominant epitope (p33) of lymphocytic choriomeningitis virus *in vitro* as observed by increased antigen-specific transgenic CD8^+^ T-cell proliferation, but with a reduced efficiency compared to peritoneal isolated DCs (59). The DCs used both the TAP-dependent and independent pathway for cross-presentation (59), but there was no difference in activation of CD8^+^ T lymphocytes between TAP1-deficient peritoneal macrophages (thioglycolate stimulated) and wild-type controls, suggesting the use of the TAP-independent vacuolar pathway (59). Finally, murine peritoneal macrophages (thioglycolate stimulated) showed efficient cross-presentation of ovalbumin encapsulated in polylactic-co-glycolic acid particles to the B3Z CD8^+^ T-cell line *in vitro*, and these particles resided in LAMP1-positive lysosomes even 48 h after phagocytosis (64). Based on this observation, it was suggested that the antigenic proteins might be translocated from the endosomal/lysosomal compartments to the cytosol. However, it might also be possible that the antigen is processed by endosomal/lysosomal proteases, and cross-presentation occurs via the vacuolar pathway.

To target cross-presentation by peritoneal macrophages, the effect of downregulation of various signaling pathways has been investigated. Compared to macrophages isolated from wild-type mice, CD11b^+^F4/80^+^ peritoneal macrophages (thioglycolate stimulated) from signal transducer and activator of transcription (STAT) 3 knockout mice were able to stimulate more potent OT-1 CD8^+^ T lymphocyte proliferation and IFN-γ production *in vitro* with irradiated tumor cells expressing ovalbumin (65). Similar observations were made with a different antigen derived from the influenza protein hemagglutinin in combination with hemagglutinin-recognizing CD8^+^ T lymphocytes isolated from CLN4 transgenic mice (65). Because STAT3 is activated by inflammatory cytokines, such as IL-6, this finding indicates that cross-presentation by peritoneal macrophages is influenced by the tissue environment. The increase in ovalbumin-recognizing CD8^+^ T lymphocyte activation was not seen for STAT4 or STAT6 knockout mice (65). Additionally, the antigen VSV8 in a complex with heat shock protein gp96 was more efficiently cross-presented (cytotoxicity by ^51^Cr release) to VSV-specific cytotoxic T cells *in vitro* by mice peritoneal macrophages compared to the antigen alone (66), again indicating that the form of the antigen plays an important role for cross-presentation by peritoneal macrophages.

So, although these results clearly show that peritoneal macrophages are able of cross-presentation *in vitro*, the *in vivo* relevance remains unclear because of the highly artificial nature of the antigen and the lacking definition of the used peritoneal macrophage subset.

## Concluding Remarks

As discussed in this review, many macrophage types seem capable of antigen cross-presentation with similar, or even better, efficiency as DCs. Moreover, macrophages seem to employ mostly the vacuolar pathway of cross-presentation, whereas DCs use both the vacuolar and cytosolic pathways. Especially the cross-presenting abilities of macrophages in the spleen, liver and lymph nodes might be physiologically relevant, because they have easy access to blood-borne antigens, whereas for the DCs, this access might be restricted. However, the role of macrophages in CD8^+^ T lymphocyte activation should be further investigated, because CD169^+^ macrophages might not activate the CD8^+^ T lymphocytes themselves but could transfer antigen to DCs by CD169 (20). The role of CD169^+^ macrophages should therefore be further investigated, and it should be investigated if they directly cross-present to CD8^+^ T lymphocytes *in vivo* or transfer the antigen to DCs (27, 29).

As apparent from this review, the *in vivo* roles and cellular pathways of macrophage cross-presentation are mostly unknown. One important reason for this is that the isolation of the specific macrophage types is technically challenging because (i) surface markers overlap resulting in contamination with other cell types, and (ii) low numbers of primary macrophages can be isolated (63). Moreover, the available *in vivo* research on cross-presentation by macrophages is almost exclusively focused on the effect of vaccination strategies, such as liposomes encapsulating antigen, where CD8^+^ T lymphocyte activation markers and/or proliferation are used as the sole measures of cross-presentation. Although CD8^+^ T lymphocyte activation and proliferation depend on cross-presentation, they also depend on other factors such as costimulation by cytokines (e.g., IL-12) and costimulatory receptors (e.g., CD80, CD86) (2). This costimulation is generally stronger present in DCs than macrophages, and it is particularly absent in the alternatively activated anti-inflammatory macrophages. Using T-cell activation as the sole readout might thereby result in overlooking cross-presentation capabilities in macrophage types that do not provide costimulation, whereas this might have important roles, for instance, in maintenance and/or restoration of immune tolerance. Cross-presentation by immature/inactivated DCs has been suggested to allow maintenance of tolerance (67), and it might well be that cross-presentation by Kupffer cells and/or anti-inflammatory macrophages plays a similar role (41, 42). Therefore, the macrophage cross-presenting capabilities and pathways should be further elucidated, for instance, *in vitro* by exposing the cells to antigens and measuring cross-presentation directly using antibodies (26, 27) followed by measuring their ability to activate CD8^+^ T-cell lines that do not need costimulation. However, because environmental factors might be needed, these *in vitro* experiments should be performed in parallel with *in vivo* experiments. To determine if a particular macrophage type is needed for efficient CD8^+^ T lymphocyte activation in *vivo*, this macrophage type could be depleted using DTR mice as previously explained (68, 69). However, this relies on surface marker expression and is not effective if the targeted macrophage type does not express a unique surface marker.

It is increasingly clear that not only DCs and macrophages can cross-present antigens, but also many other endocytic cell types are capable of cross-presentation, including monocytes (70, 71), B cells (72), neutrophils (73), and endothelial cells (74). The physiological roles of cross-presentation by these diverse cell types are still unclear, but it seems likely that this also allows the potentiation of CD8^+^ T lymphocyte responses or the maintenance or restoration of immune tolerance.

Lastly, studies on cross-presentation have focused on direct CD8^+^ T lymphocyte activation, but the potential role of tissue macrophages in development and reactivation of memory CD8^+^ T lymphocytes has hardly been investigated. Although CD11c^int^F4/80^high^ splenic red pulp macrophages are not essential for development of memory CD8^+^ T lymphocytes (23), it might well be that other macrophage types are involved in this process or that macrophages are capable of reactivation of memory CD8^+^ T lymphocytes upon recurrent immune challenges (75, 76). Recently, there is a growing interest in the activation of memory T cells in the tissues and lymphatic system, which can confer rapid host protection upon cognate antigen-mediated activation and results in direct killing of infected cells (75, 76). Especially tissue-resident macrophages might be important in the development and reactivation of tissue-resident memory CD8^+^ T lymphocytes by rapid and local cytokine secretion during re-infection (74). It is increasingly clear that macrophages can provide factors such as chemokines and cytokines that regulate the localization, differentiation, and survival of tissue-resident memory T cells (73). Moreover, monocyte-derived APCs provide TNF superfamily costimulatory signals, which substantially increase the formation of tissue-resident memory T cells during viral infection (73).

Because macrophages can cross-present and thereby might aid in CD8^+^ T lymphocyte responses, stimulating macrophages to cross-present might be a promising strategy for antitumor or antiviral therapies.

## Author Contributions

EM and LM wrote the manuscript and GB participated in discussion and reviewed/edited the manuscript. All authors contributed to the article and approved the submitted version.

## Conflict of Interest

The authors declare that the research was conducted in the absence of any commercial or financial relationships that could be construed as a potential conflict of interest.
